# Successful Interventional Radiology for Acute Median Arcuate Ligament Syndrome After Pancreaticoduodenectomy

**DOI:** 10.7759/cureus.13540

**Published:** 2021-02-24

**Authors:** Takehiko Hanaki, Teruhisa Sakamoto, Shinsaku Yata, Yuki Murakami, Yoshiyuki Fujiwara

**Affiliations:** 1 Department of Gastroenterological Surgery, Tottori University, Faculty of Medicine, Yonago, JPN; 2 Division of Radiology, Tottori University, Faculty of Medicine, Yonago, JPN

**Keywords:** median arcuate ligament syndrome, celiac artery compression syndrome, acute-onset, pancreaticoduodenectomy, interventional radiology

## Abstract

Background: Median arcuate ligament (MAL) syndrome (MALS), also known as celiac artery (CA) compression syndrome and Dunbar syndrome, occurs because of extraluminal compression of the CA root by the MAL, which is part of the diaphragm. In MALS, a malposition of the MAL compresses the CA and causes nonspecific symptoms, including epigastric pain after eating, weight loss, nausea, and vomiting and can sometimes cause visceral aneurysms. Typically, in MALS, various chronic ischemic symptoms and visceral aneurysms due to changes in arterial blood flow are observed; however, in acute-onset MALS, acute organ failure due to ischemic changes may be problematic. Surgical treatment is the recommended treatment for MALS, but the optimal treatment of acute MALS that occurs after laparotomy remains controversial because of its rarity. Here, we present the first case of acute MALS, which occurred after pancreaticoduodenectomy (PD) that was successfully treated with interventional radiology (IVR) without reoperation.

Case presentation: A 75-year-old man presented with liver infarction after subtotal stomach-preserving PD using the Child method plus Braun enteroenterostomy. As a result of contrast-enhanced computed tomography for the investigation of elevated hepatic cytolysis-related enzymes on the first postoperative day, he was diagnosed with acute MALS resulting from gastrointestinal reconstruction after PD. The patient underwent IVR to restore blood flow of the CA, and an intraluminal stent was inserted. Despite the development of ischemic gastropathy, splenic infarction, and pancreatic fistula, the patient was eventually discharged on postoperative day 82 without any disability.

Conclusion: Many studies have reported open, laparoscopic, and robot-assisted MAL incisions for MALS, but few reports have detailed the treatment for postoperative MALS. Here, we report the first case of acute MALS developed after PD that was successfully treated with endovascular CA stenting. For acute MALS after PD, early endovascular treatment may be more useful than re-laparotomy.

## Introduction

Because median arcuate ligament (MAL) syndrome (MALS), also called “celiac axis compression syndrome” [1,2], may be fatal if left untreated at the time of pancreaticoduodenectomy (PD) [3,4], it is diagnosed preoperatively and treated with preoperative interventional radiology (IVR) or intraoperative surgical treatment. However, MALS can also occur postoperatively, even if the diagnosis of MALS is not based on preoperative or intraoperative evaluation [3,5,6]. Here, we present the first case of acute MALS, which occurred after PD that was successfully treated with IVR without reoperation.

## Case presentation

**Figure 1:**
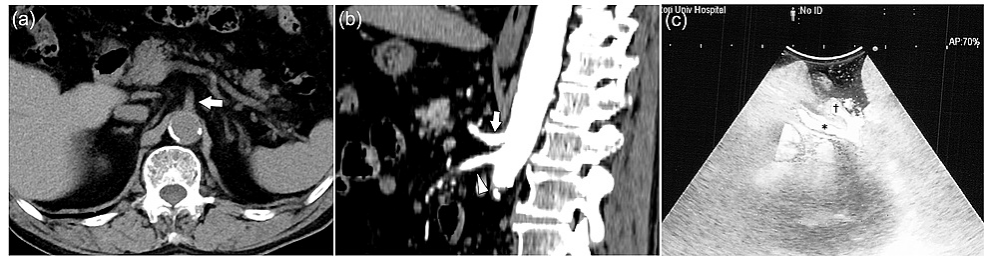
(a) Preoperative plain CT showing no arteriosclerosis around the celiac artery (CA) trunk (arrow). (b) Preoperative contrast-enhanced CT showing no evidence of median arcuate ligament syndrome (MALS). (sagittal view; arrow shows the CA; arrowhead shows the superior mesenteric artery) (c) Intraoperative ultrasonography showing good hepatic arterial blood flow. (* right hepatic artery, † left hepatic artery)

A 75-year-old asymptomatic Asian man presented to our surgical department with a cystic lesion in the pancreatic head. His medical history included hypertension and diabetic mellitus, and his body mass index was 25.8 kg/m^2^ (body weight, 63.4 kg; height, 158.0 cm), with predominantly visceral fat. Abdominal computed tomography (CT) revealed a multilocular cystic lesion with a contrast-enhanced nodule in the pancreatic head; no arterial calcification from the aorta to the celiac artery (CA) trunk was noted (Figure 1a).

**Figure 2:**
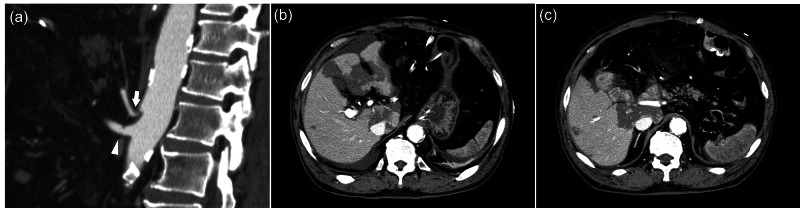
Contrast-enhanced CT on postoperative day 1 (a) Sagittal view of the arteries (arrow, “hook”-shaped celiac artery (CA); arrowhead, superior mesenteric artery). Note that the superior mesenteric artery is also shifted cranially compared with that of the preoperative CT. (b) Segmental liver infarction can be seen. In the hilar area of the liver, the hepatic artery shows decreased blood flow. (c) Splenic infarction is also confirmed.

No stenosis or flexion of the CA was noted on preoperative contrast-enhanced CT, which was not suggestive of MALS (Figure 1b). The preoperative relevant laboratory data were within normal limits except the hemoglobin A1c level (8.4%, reference range <5.6%). The systemic atherosclerotic changes were age-appropriate, and blood tests revealed normal triglyceride and cholesterol levels. We diagnosed the cystic lesion as a branch-duct type intraductal papillary mucinous neoplasm with “high-risk stigmata” and elected to perform subtotal stomach-preserving PD. The laparotomy was performed with a midline abdominal incision, and no major deformities were found in the vascular tract. No major anatomical changes were observed during surgery. Prior to ligation, we performed a clamp test of the gastroduodenal artery, and hepatic arterial blood flow was good under clamping. Subtotal stomach-preserving PD was performed as planned, and a modified Child's reconstruction was performed. After surgery, blood flow in the hepatic artery was confirmed by Doppler ultrasonography (Figure 1c), and the abdominal cavity was closed. After returning to the intensive care unit, the patient's vital signs were stable, but his lactate level seemed to decline more slowly than usual. Hepatic cytolysis-related enzymes (aspartate aminotransferase, 867 IU/L; alanine transaminase, 707 IU/L) were elevated on postoperative day (POD) 1, and a contrast-enhanced CT was performed because of concerns of decreased blood flow to the liver. Emergency CT revealed stenosis of the CA and angular change in relation to the aorta of the CA trunk (Figure 2a), which were believed to have caused decreased CA blood flow (Figure 2b, 2c).

**Figure 3:**
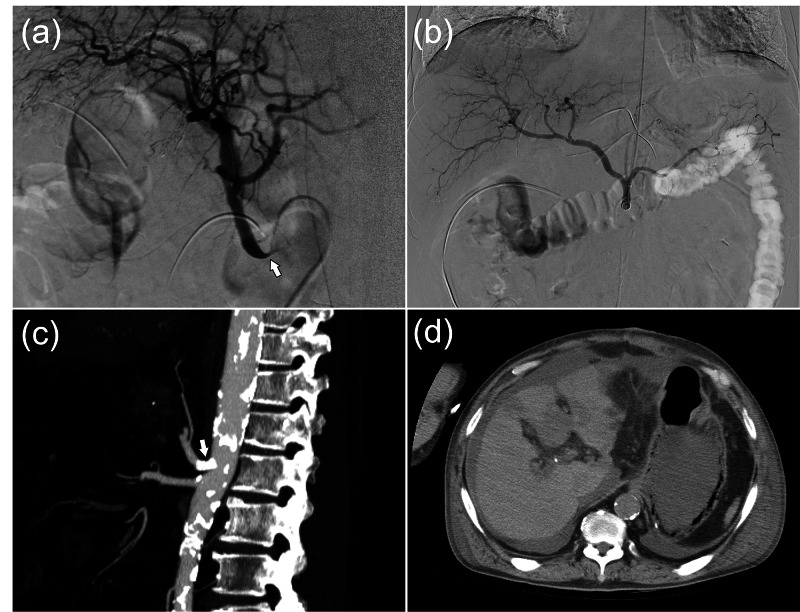
(a) Emergency arteriography showing flexion of the celiac artery (CA) root (the arrow, CA; lateral view). Subsequently, measurement of the CA pressure demonstrated a pressure gradient before and after the stenotic area. (b) Celiac arteriography after bare metal stent insertion. The stenosis was then released. (c) Contrast-enhanced CT on postoperative day (POD) 7 shows CA patency and improved CA blood flow. The arrow indicates the bare metal stent inserted into the CA. (d) Plain CT from POD7 shows gastric emphysema as a change associated with ischemic gastropathy.

The patency of the portal vein and its intrahepatic branches was normal. On the basis of these results, we decided to perform IVR to improve blood flow in the CA on POD1. On celiac arteriography, CA stenosis was revealed (Figure 3a), and the catheter was inserted from the CA to the common hepatic artery with ease, which indicates that the CA was not mechanically obstructed but had stenosis due to flexure.

A pressure gradient (approximately 60 mmHg) was observed before and after the stenosis, and it was judged that the narrowing of the CA lumen was not due to a change in the lumen but was caused by flexion of the CA. These findings led to the diagnosis of acute MALS associated with post-PD gastrointestinal reconstruction. Via left brachial artery access using a 6-Fr sheath, a bare metal stent (6 × 14 mm, Express SD^TM^, Boston Scientific, Marlborough, MA, USA) was inserted into the CA to improve blood flow in the flexure, and the procedure was completed after improved blood flow to the liver was confirmed by arteriography (Figure 3b). After IVR, antiplatelet agents (clopidogrel, 75 mg/day/body) and anticoagulant therapy (heparin, 10000 U/day/body) were introduced, after which CA restenosis and flexion did not occur (Figure 3c). The patient did not develop a liver abscess, bile fistula, or cholangitis, and the infarcted areas in the liver and spleen gradually disappeared. Intramural emphysema of the gastric wall, as a change in ischemic gastropathy, was observed incidentally on CT on POD7 (Figure 3d), but the emphysema resolved over the next week.

Prompt IVR for CA flexion was successful in the prevention of hepatic failure. The patient required a longer hospital stay than usual due to pancreatic fistula (grade B; International Study Group of Pancreatic Fistula [7]). He was eventually discharged on POD82 with no disability or organ failure with continuation of the antiplatelet therapy. The stent required permanent implantation, and the patient had survived without stent occlusion for six months after discharge.

## Discussion

In 1917, Lipshutz first described MAL compression of the CA from an anatomical perspective [8], and the symptoms of MAL compression of the CA trunk, known as MALS, were first reported in 1963 by Harjola and in 1965 by Dunbar [9,10]. Although MALS is very rare, with a reported incidence of 0.002% in the total population [11], asymptomatic MAL compression of the CA reportedly occurs in 7.3%-33% of cases; however, asymptomatic MALS is considered nonpathogenic [12,13]. Two explanations may explain the cause of the symptoms of MALS [14,15]. The first is the mesenteric ischemia caused by MAL compression of the CA, and the second is the direct irritation and compression of the celiac ganglion or plexus by the MAL. In any case, changes caused by non-acute MALS are mainly subjective in nature. In contrast, in cases of acute-onset MALS, such as the present case, organ damage occurs as a result of impaired CA blood flow. Actually, ischemic changes in the liver, spleen, and stomach were noted on imaging in the presented case. Although it is difficult to determine the association with ischemia, leakage at the pancreaticojejunostomy probably occurred because of ischemia of the pancreas, which is perfused by the CA. The pancreatic head contains several arterial arcades that connect the CA to the superior mesenteric artery, and CA stenosis does not usually cause organ perfusion disturbance or organ failure. However, after PD, acute perfusion disorders of the CA-perfused area may occur because of MALS because PD eliminates the arterial arcade in the pancreatic head. In the present case, we believe that loss of the pancreatic head artery arcade after PD and movement of the small intestinal mesentery with abundant visceral fat to above the hepatic hilum for gastrointestinal reconstruction in the presence of MAL were the causes of acute MALS (Figure 2a). This could not be diagnosed preoperatively or intraoperatively, but if it had been diagnosed, a MAL incision should have been performed. Because it is very difficult to predict postoperative hepatic arterial hypoperfusion either preoperatively or intraoperatively in such a case, it is crucial to detect and treat this condition soon after surgery.

We performed a PubMed search and revealed three previous cases of acute-onset MALS after PD, which are summarized in Table 1.

**Table 1:** Review of the literature: case reports of acute median arcuate ligament syndrome after PD PD, pancreaticoduodenectomy; MAL, median arcuate ligament; MALS, median arcuate ligament syndrome; BMI, body mass index; PDAC, pancreatic ductal adenocarcinoma; PPPD, pylorus-preserving PD; POD, postoperative day; IPMN, intraductal papillary mucinous neoplasm; SSPPD, subtotal stomach-preserving PD; IVR, interventional radiology; n.d., not described

No.	Year	Author	Sex	Age (years)	BMI (kg/m^2^)	Preoperative diagnosis for PD	Operation method	Reconstruction method	Preoperative diagnosis of MALS	Date of MALS diagnosis	Ischemic organ	Treatment	Complication	Date of discharge
1	2013	Sanchez et al. [5]	Female	73	n.d.	PDAC	PPPD	n.d.	No	POD2	Liver	Conservative	Uneventful	POD26
2	2016	Karabicak et al. [6]	Male	40	n.d.	PDAC	PD	n.d.	No	POD1	Liver	MAL incision	Bile leakage from hepaticojejunostomy	POD43
3	2017	Imai et al. [3]	Female	69	n.d.	PDAC	PD	n.d.	No	POD3	Liver, spleen, stomach, and remnant pancreas	IVR, MAL incision, remnant pancreatectomy, splenectomy, and percutaneous transhepatic cholangiodrainage	Necrosis of pancreaticojejunostomy and remnant pancreas, rupture of gastrojejunostomy, and stenosis of choledocojejunostomy	POD216
4	2020	Present case	Male	75	25.8	IPMN	SSPPD	Child method	No	POD1	Liver, spleen, stomach, and remnant pancreas	IVR	Pancreatic fistula	POD82

Sanchez et al. reported a case of MALS with liver ischemia that improved with conservative follow-up [5]; the other reported cases of MALS underwent reoperation using a MAL incision [3,6]. In non-acute-onset MALS, surgical MAL incision would be more desirable than endovascular treatment with IVR or arterial bypass [2], because re-laparotomic procedures after PD would be difficult because of adhesions and effects on the reconstructed organs. Imai et al. performed IVR prior to MAL incision, but they eventually performed a second surgery because of CA re-occlusion, which resulted in a third surgery, remnant pancreatectomy, and splenectomy [3]. That report differs from this case in the time of diagnosis and IVR treatment. Imai et al. reported that MALS was diagnosed on POD3. In contrast, in this case, the patient was diagnosed with MALS on POD1, and IVR treatment was performed soon after the diagnosis. This indicates the importance of early diagnosis and treatment.

Patency of the CA after PD is a crucial factor, and if compromised, catastrophic hepatic, biliary, splenic, pancreatic, and gastric ischemia can occur [3,4]. This is the fourth report of a decrease in hepatic arterial blood flow in MALS that was not evident at the time of intraoperative gastroduodenal artery clamping or at the time of abdominal closure and the first report of successful IVR immediately after a MALS diagnosis. Surgeons specializing in hepatobiliary and pancreatic surgery should be aware that MALS can occur acutely after PD, even if it is not diagnosed preoperatively or intraoperatively.

## Conclusions

To our best knowledge, we reported the first case of acute post-PD MALS that was successfully treated with prompt stent interpolation for CA flexion to prevent hepatic failure. For acute MALS after PD, early endovascular treatment may be more useful than re-laparotomy.
